# 

**Published:** 1884-11

**Authors:** 


					﻿Vol. IV.
NOVEMBER, 1884.
NO. 11.
THE
pOMCEOPATHlC pHY^lClAJfl,
A MONTHLY JOURNAL OF MEDICAL SCIENCE.
“Seek the Truth: come whence it may, cost what it will.”
ZE. J\ LHE, HVE. ID.,
EDITOR.
CONTENTS:
(See inside of Cover.)
PHILADELPHIA:
No. 129 SOUTH THIRTEENTH STREET.
NEW YOBB;:
A. L. CHATTERTON, PUBLISHING COMPANY, PUBLISHER’S AGENTS.
T, O 25T ID O 3ST :
ALFRED HEATH & CO., Sole Agents for Great Britain,
114 Ebury Street, Eaton Square.
Yearly Subscription, $2.50, invariably in advance.
Entered at the Philadelphia Post-Office as Second- Class Mail Matter.
				

